# YY2 Serves as a Novel Prognostic Biomarker Correlated with Immune Microenvironment and Glycolysis in Esophageal Carcinoma

**DOI:** 10.2174/0113892029358348250124064940

**Published:** 2025-02-08

**Authors:** Haimei Gou, Hui Yang, Jiao Cheng, Shuang He, Can Luo, Xin Chen, Xiaowu Zhong

**Affiliations:** 1 Department of Clinical Laboratory, Affiliated Hospital of North Sichuan Medical College, Nanchong, 637000 Sichuan, China;; 2 School of Laboratory Medicine, North Sichuan Medical College, Nanchong, 637000 Sichuan, China;; 3 Translational Medicine Research Center, North Sichuan Medical College, Nanchong, 637000 Sichuan, China;; 4 Department of Rehabilitation Medicine, Affiliated Hospital of North Sichuan Medical College, Nanchong, 637000 Sichuan, China

**Keywords:** Esophageal carcinoma, YY2, prognosis, glycolysis, immune microenvironment, biomarker

## Abstract

**Background:**

Yin Yang 2 (YY2) plays a pivotal role in various tumorigenic processes; however, its specific involvement in esophageal carcinoma (ESCA) remains elusive. This study aims to investigate the expression and potential functional significance of YY2 in ESCA.

**Methods:**

The expression and functions of YY2 in ESCA were analyzed using a broad range of bioinformatics databases and tools, including TCGA, TIMER, TISIDB, QUANTISEQ, cBioPortal, DNMIVD, LinkedOmics, DAVID, GSEA, GEPIA2, LASSO, miRWalk, miRDB, and TargetScan. Furthermore, RT-qPCR, immunohistochemical staining, western blot, CCK8 assay, and wound healing assay were employed to validate the involvement of YY2 in ESCA pathogenesis.

**Results:**

Bioinformatics analyses revealed that the YY2 gene is upregulated in ESCA tissues, with its high expression significantly associated with poor prognosis and elevated levels of M2 macrophages, NK cells, Tregs, CTLA4, TIGIT, and Siglec-15. Validating the ESCA samples demonstrated that knockdown of YY2 effectively inhibited cell proliferation and migration in ESCA cells. The biological functions of YY2 and its co-expressed genes were primarily associated with transcriptional regulation, DNA methylation, glycometabolism, and ubiquitination. Moreover, the regulatory network of YY2 in the glycolysis pathway was found to involve multiple genes and miRNAs. Finally, a prognostic model based on YY2 and its associated glycolysis genes revealed a strong inverse correlation between higher risk scores and lower survival rates in esophageal adenocarcinoma (EAC).

**Conclusion:**

YY2 may serve as a promising prognostic biomarker and an innovative therapeutic target for patients with ESCA, regulating cell proliferation, migration, immune microenvironment, and glycolysis.

## 1 INTRODUCTION

According to the Global Cancer Statistics 2022 database, esophageal carcinoma (ESCA) ranks eleventh in incidence and seventh in mortality among all cancer types [1]. Notably, China accounts for approximately 50% of global ESCA incidence and deaths [2, 3]. ESCA is primarily classified into two major histological subtypes: esophageal adenocarcinoma (EAC) and esophageal squamous cell carcinoma (ESCC). Early diagnosis of ESCA is challenging, and the prognosis remains poor. Despite advancements in treatment strategies, the five-year survival rate remains approximately 15% to 25% [4, 5]. Therefore, it is imperative to elucidate the mechanisms underlying ESCA occurrence and progression, as well as to identify novel diagnostic and therapeutic targets to improve patient outcomes.

Yin Yang 2 (YY2) and Yin Yang 1 (YY1) are multifunctional zinc finger proteins belonging to the Yin Yang (YY) family. These proteins function as transcriptional activators and repressors of their target genes [6, 7]. YY2 is derived from YY1 mRNA and shares more than 50% homology in DNA and amino acid sequences. Initially, YY2 was believed to solely assist YY1 in regulating target genes [8]. Evidence suggests that YY2 and YY1 exhibit overlapping functions in cardiovascular development [6]. However, accumulating data indicate that YY2 plays distinct roles beyond redundancy with YY1, particularly in cardiovascular and nervous systems, as well as in embryonic stem cell growth [9-13]. Moreover, recent studies have demonstrated competitive binding between YY2 and YY1 to identical DNA binding sites, leading to antagonistic regulation of ferroptosis in tumor cells [7]. The proposed functions of YY2 generally involve redundancy or antagonism relative to YY1.

YY1 exhibits dual roles as a tumor suppressor and an oncogene, with aberrant expression detected in the majority of tumors [14-19]. It regulates diverse molecular mechanisms, including transcriptional regulation and epigenetic modification [14-19]. Some studies suggest that YY2 may function as a potential tumor suppressor gene [7, 20-23]. However, the precise role of YY2 in tumorigenesis remains unclear. Whether YY2 acts as a tumor promoter or suppressor in ESCA remains to be explored as currently, no studies have examined its involvement in ESCA.

In this study, the expression, genetic alterations, methylation status, immune microenvironment, co-expressed genes, and functional annotation of YY2 in ESCA patients were analyzed using online databases. Additionally, a comprehensive analysis of the regulatory network involving YY2 in the glycolysis pathway in ESCA was performed, and a prognostic signature model based on YY2 and related glycolysis genes was constructed. Furthermore, YY2 expression was validated in ESCA samples through RT-PCR and immunohistochemical staining. Our results confirm for the first time that YY2 functions as a tumor promoter in ESCA through knockdown experiments in ESCA cell lines. These findings suggest that YY2 may serve as a prognostic biomarker and potential therapeutic target for ESCA treatment.

## 2 MATERIALS AND METHODS

### 2.1 Gene Expression and Clinical Characteristics Analysis

The TIMER (https://cistrome.shinyapps.io/timer/) [24] database was used to analyze YY2 mRNA expression profiles in pan-cancer tissues and their corresponding adjacent normal controls across all TCGA tumors. RNA expression data were retrieved from the TCGA (https://www.cancer.gov/tcga) and GTEx (https://gtexportal.org/home/datasets) databases. Expression levels of YY2 mRNA were compared between ESCA tumors and normal tissues retrieved from TCGA, further corroborated by integration with GTEx data. The relationship between YY2 expression and clinicopathological characteristics in ESCA patients was examined using LinkedOmics (http://www.linkedomics.org/login.php) [25].

### 2.2 Genetic Alterations and Methylation Analysis

The cBioPortal (http://www.cbioportal.org/) [26] was used to investigate genetic alterations and their impact on overall survival in 368 ESCA samples from two datasets: TCGA Firehose Legacy and TCGA PanCancer Atlas. The DNMIVD database (http://1193.41.228/dnmivd/) [27] was utilized to analyze the methylation status of the YY2 promoter region and its correlation with YY2 expression in ESCA.

### 2.3 Immune Microenvironment Analysis

The TIMER database, quanTIseq algorithm, and TISIDB database were employed to evaluate the immune status in ESCA. The TIMER database was used to investigate correlations between YY2 expression and immune cell infiltration in ESCA. Using the R software package “immunedeconv” and the quanTIseq algorithm, differences in immune cell profiles between high and low YY2 expression groups within the ESCA TCGA cohort were analyzed. Relationships between chemokines, immunoinhibitors, immunostimulators, MHCs, and YY2 expression were explored using TISIDB (http://cis.hku.hk/TISIDB/) [28]. Additionally, the expression levels of immune checkpoint-related genes were compared between high and low YY2 expression groups within the ESCA TCGA cohort.

### 2.4 Co-expression Analysis of the YY2 Gene and its Putative Functions

The LinkedOmics platform was employed to analyze co-expressed genes associated with YY2 transcription in ESCA. The biological functions of these co-expressed genes were further evaluated using the DAVID database (https://david.ncifcrf.gov/) [29], which encompasses biological processes (BP), molecular functions (MF), cellular components (CC), and KEGG pathways.

### 2.5 Analysis of the Glycolytic Pathway Associated with YY2 in ESCA

The gene set associated with the YY2 glycolytic pathway was determined by identifying the intersection between co-expressed genes and glycolysis-related gene sets. Nine glycolysis-related MSigDB gene sets were retrieved from GSEA (http://www.gsea-msigdb.org/gsea/index.jsp), including BIOCARTA_GLYCOLYSIS_PATHWAY, GOBP_ GLYCOLYTIC_PROCESS, HALLMARK_GLYCOLYSIS, HP_ABNORMALITY_OF_GLYCOLYSIS, KEGG_GLYCO LYSIS_GLUCONEOGENESIS, REACTOME_GLYCOLY SIS, WP_GLYCOLYSIS_AND_GLUCONEOGENESIS, WP_GLYCOLYSIS_IN_SENESCENCE, and WP_HIF1A_ AND_PPARG_REGULATION_OF_GLYCOLYSIS. To identify miRNAs interacting with YY2, we utilized the miRWalk (http://mirwalk.umm.uni-heidelberg.de/), miRBD (https://mirdb.org/), and TargetScan (https://www.targetscan.org/vert_80/) databases to select a consensus from these three databases. A glycolytic pathway network diagram involving miRNA, YY2, and co-expressed genes was constructed using Cytoscape 3.7.2.

### 2.6 Prognostic Analysis

The GEPIA platform (http://gepia.cancer-pku.cn/index) [30] was utilized to investigate the association between gene expression levels and prognosis, as well as to generate survival curves for Overall Survival (OS) and Disease-Free Survival (DFS). LASSO regression analysis using the R software glmnet package [31] was performed on YY2 and its associated glycolytic genes within the ESCA cohort of TCGA to refine the pool of candidate genes. A multivariable Cox regression analysis was conducted using the 'survival' R package [32, 33] to construct a prognostic model. Kaplan-Meier (KM) survival analysis was employed to assess differences in survival between high-risk and low-risk score groups, while time-dependent ROC analysis was used to evaluate the predictive accuracy of the model.

### 2.7 Human Tissue Samples

The study included 32 pairs of ESCA tissues with pathological diagnoses and their corresponding adjacent normal tissues, which were obtained from the affiliated hospital of North Sichuan Medical College. The study strictly adhered to the principles outlined in the Helsinki Declaration. This study was approved by the Medical Ethics Committee of the Affiliated Hospital of North Sichuan Medical College (approval No. k2020009), and all participating patients provided informed consent.

### 2.8 Real-time PCR

Total RNA was isolated from tissues using TRIzol (Ambion, Carlsbad, USA), followed by reverse transcription into cDNA using the Transcriptor First-Strand cDNA synthesis kit (Roche, Basel, USA). Subsequently, RT-qPCR was performed on a LightCycler 480 system (Roche, Basel, USA) with Fast SYBR Green Master mix (Takara, Tokyo, Japan). The relative expression of YY2 was analyzed using the 2-∆∆Ct method with normalization to GAPDH. The experiment was conducted in triplicate. The RT-qPCR primer sequences are listed in Table **S1**.

### 2.9 Western Blot

Protein was extracted using RIPA lysis buffer supplemented with protease and phosphatase inhibitors (Beyotime, Shanghai, China). Protein concentration was quantified using a BCA kit (Thermo Fisher, Massachusetts, USA). Proteins were separated by 12% SDS-PAGE and transferred onto polyvinylidene fluoride (PVDF) membranes (Millipore, Billerica, USA). After blocking with 5% skim milk in tris-buffered saline with Tween-20 (TBST) for 1 hour, membranes were incubated overnight at 4°C with YY2 antibody (catalog number: sc-374455, dilution: 1:500; Santa Cruz Biotechnology, CA, USA) [34-36] or GAPDH antibody (catalog number: HA721136, dilution: 1:2000; HUABIO, Hangzhou, China). Membranes were then incubated at room temperature for 1 hour with a secondary antibody. Protein bands were visualized using an ECL reagent (Millipore, Billerica, USA) and detected using an imaging system (Vilber, Paris, France). Each experiment was performed in triplicate.

### 2.10 Immunohistochemical Staining

YY2 protein expression in ESCA tissues was detected using an immunohistochemical (IHC) staining kit (ZSJQ BIO, Beijing, China) following the manufacturer's instructions. The antibodies used included the YY2 antibody (catalog number: sc-374455, dilution: 1:100, Santa Cruz Biotechnology, CA, USA) and secondary antibodies. The pathological results were evaluated by two senior chief pathologists. Yellow or brown granular substances were observed in the slices; staining intensity was scored as 0 for no staining, 1 for light yellow (weakly positive), 2 for brown (moderately positive), and 3 for deep brown (strongly positive). Tumor cell proportion was scored as follows: 0 for no positive cells, 1 for <25% positive cells, 2 for 25%~50%, 3 for 50%~75%, and 4 for ≥75% positive cells. The final staining index was obtained by multiplying the percentage of positive cells by the staining intensity score.

### 2.11 Transient Transfection

ECA109 and TE1 cells were selected for functional investigation. Plasmids encoding YY2 knockdown (sh-YY2) and corresponding controls (sh-NC) were synthesized by Hanbio (Hanbio, Shanghai, China). Transfection was performed using Lipofectamine 2000 reagent (Invitrogen, Waltham, MA, USA) according to the manufacturer’s protocol. The shRNA sequences are listed in Table **S1**.

### 2.12 CCK8 Assay

Single-cell suspensions of ECA109 and TE-1 cells were prepared using trypsin digestion, with the concentration adjusted to 5×10^4^ cells/mL. Subsequently, 100 µL of the suspension was added per well to a 96-well plate and cultured for 24 hours. Four parallel groups were established at the following time points: 0 h, 24 h, 48 h, and 72 h. At each time point, cells were treated with 10 µL CCK8 reagent (Beyotime, Shanghai, China) per well. After 2 hours of incubation in a cell culture incubator, absorbance was measured at a wavelength of 450 nm.

### 2.13 Wound Healing Assay

ECA109 and TE-1 cells in the logarithmic phase were transferred to a six-well plate. When cell density exceeded 90%, a 10 µL pipette tip was used to create a straight scratch along a sterile ruler on the cell-covered plate. DMEM culture medium with 2% FBS (Gibco, USA) was then added for further cultivation. The width of the scratch at the same position was observed and measured under a microscope at 0 h and 48 h. Three random photographs were taken and quantified.

### 2.14 Statistical Analysis

RNA expression data from databases were analyzed using R 4.0.3 The outcomes of RT-qPCR and IHC staining were analyzed using SPSS 19.0. GraphPad Prism 8.0 was utilized for figure generation. Student's t-test, or the Wilcoxon test, was conducted to assess differences between the two groups. Correlations were evaluated using Pearson’s correlation analysis. Statistical significance was defined as *P < 0.05, **P < 0.01, or ***P < 0.001.

## 3 RESULTS

### 3.1 YY2 is Upregulated in ESCA

**Fig. (1):**
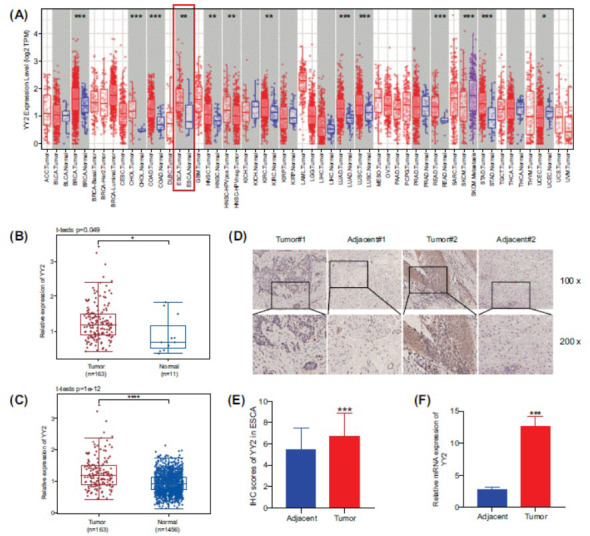
Elevated expression of YY2 in ESCA. (**A**) The expression levels of YY2 across various cancer types based on TCGA data. (**B** and **C**) Upregulation of YY2 expression in ESCA tissues compared to normal tissues in the TCGA and GTEx+TCGA datasets. (**D**) Representative IHC images illustrating YY2 staining in ESCA tumors and adjacent tissues (scale bar: 100 μm; magnification: 100× and 200×). (**E**) IHC scores of YY2 in ESCA tissues and matched adjacent tissues. (**F**) mRNA expression levels of YY2 in ESCA tissues and matched adjacent tissues. *P<0.05, **P<0.01, ***P<0.001.

The TIMER analysis demonstrated a significant upregulation of YY2 expression in various tumor types, including ESCA, bile duct cancer, and rectal cancer, compared to normal tissues (Fig. **1A**). This upregulation was confirmed in ESCA based on TCGA-ESCA data (Fig. **1B**). Additionally, the integration of GTEx and TCGA samples further validated the increased expression levels of YY2 in ESCA (Fig. **1C**). Subgroup analyses revealed elevated YY2 expression levels specifically in ESCC and EAC (Fig. **S1**). However, LinkedOmics analysis did not identify any significant correlations between YY2 expression and other clinical parameters, such as age, race, pathological grade, pathological T stage, pathological N stage, or pathological M stage, in TCGA samples (Table **S2**).

RT-qPCR and IHC staining were performed to assess the expression of YY2 in tissues from ESCA patients and their corresponding adjacent tissues. The results revealed significantly higher YY2 mRNA levels (Fig. **1F**) and protein expression (Fig. **1D** and **E**) in ESCA tissues compared to adjacent tissues. These findings provide compelling evidence for the elevated presence of YY2 in ESCA tissues.

### 3.2 Genetic Alterations and Methylation Status of YY2 in ESCA

**Fig. (2):**
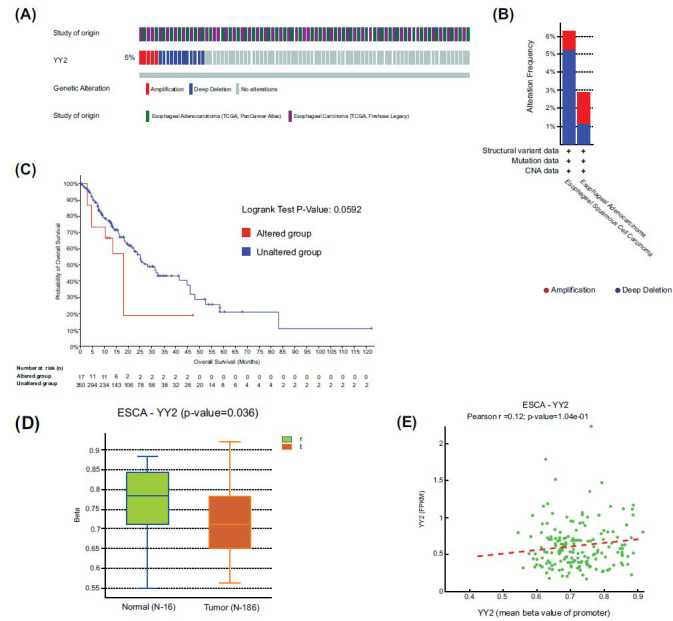
Genetic alterations and methylation status of YY2 in ESCA. (**A**) OncoPrint visual summary of YY2 alterations in ESCA. (**B**) Mutation rates of YY2 in ESCC and EAC. (**C**) Survival analysis of YY2 alterations in ESCA. (**D**) DNA methylation levels of YY2 in ESCA tissues compared to normal tissues. (**E**) Correlation between mRNA expression and methylation status of YY2 in ESCA.

Gene mutation analysis using the cBioPortal database revealed a mutation rate of approximately 5% for YY2 in ESCA, with amplification and deletion being the predominant mutation forms (Fig. **2A**). A higher mutation rate was observed in ESCC compared to EAC (Fig. **2B**). Survival analysis indicated that individuals with altered YY2 demonstrated a trend towards poorer prognosis, with borderline significance (*p* = 0.0592, Fig. **2C**). Further analysis using the DNMIVD database showed significantly lower methylation levels in the promoter region of YY2 in ESCA tissues compared to normal tissues (Fig. **2D**). However, no statistically significant correlation was observed between YY2 expression levels and methylation levels (Fig. **2E**).

### 3.3 YY2 and the Immune Microenvironment in ESCA

**Fig. (3):**
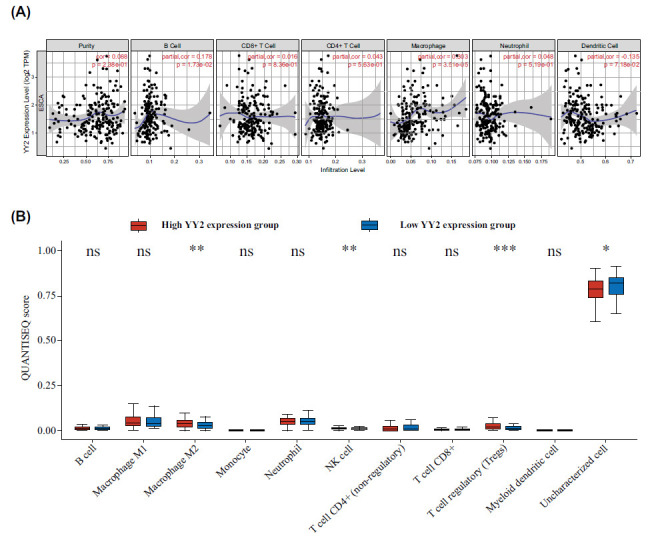
Relationship between YY2 and immune cells in ESCA. (**A**) Correlation between YY2 expression and immune cell infiltration, based on the TIMER database. (**B**) Immune cell scores in ESCA with high and low YY2 expression determined using the QuanTIseq algorithm. *P<0.05, **P<0.01, ***P<0.001, ^ns^*P*≥0.05.

The TIMER analysis revealed a significant positive correlation between YY2 expression and immune cell infiltration, specifically with macrophages (*P* = 3.51e−05) and B cells (*P* = 1.73e−02) (Fig. **3A**). Utilizing the quanTIseq algorithm within the TCGA-ESCA cohort, elevated levels of M2 macrophages, NK cells, and Tregs were identified in the high YY2 expression group (Fig. **3B**), indicating a positive association between YY2 expression and these immune cell populations.

**Fig. (4):**
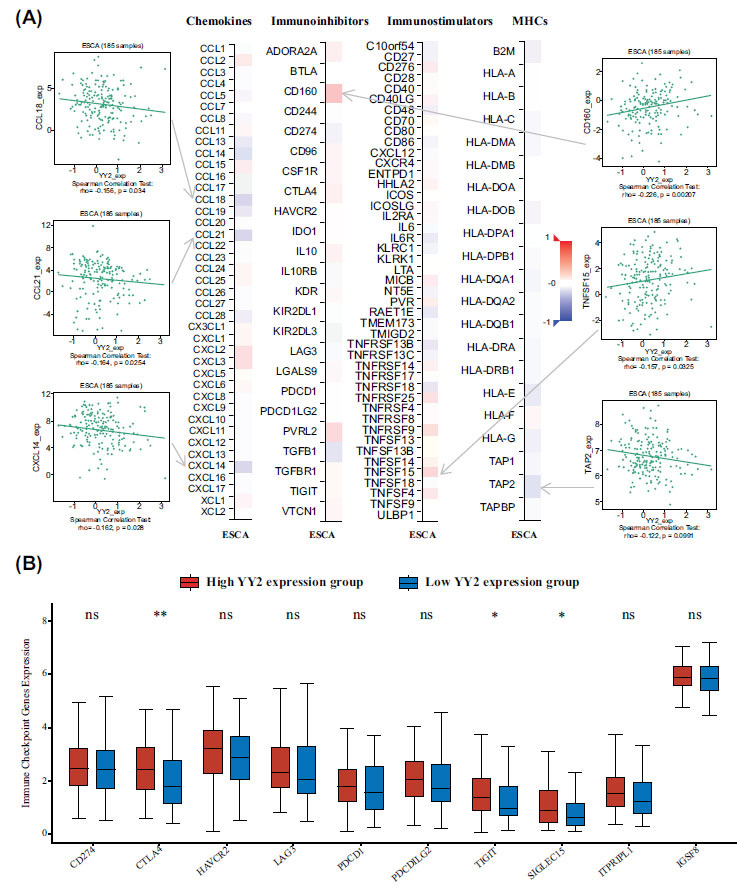
Relationship between YY2 and immune mediators or immune checkpoints in ESCA. (**A**) Correlation between YY2 expression and immune mediators, as shown in the TISIDB database. (**B**) Expression of immune checkpoint-related genes in ESCA with high and low YY2 expression. *P<0.05, **P<0.01, ^ns^*P*≥0.05.

The relationship between YY2 expression and immune-related factors was further examined using the TISIDB database. YY2 expression exhibited negative correlations with chemokines, such as CCL18, CCL21, and CXCL14, while displaying positive correlations with the immunoinhibitor CD160 and the immunostimulator TNFSF15 (all *P* < 0.05, Fig. **4A**). Additionally, YY2 expression negatively correlated with the MHC molecule TAP2. Analysis of immune checkpoint markers in TCGA-ESCA samples revealed that high YY2 expression was associated with elevated expression of immune checkpoints, including CTLA4, TIGIT, and Siglec-15 (all *P* < 0.05, Fig. **4B**).

### 3.4 Co-expression Network and Putative Function of YY2 in ESCA

**Fig. (5):**
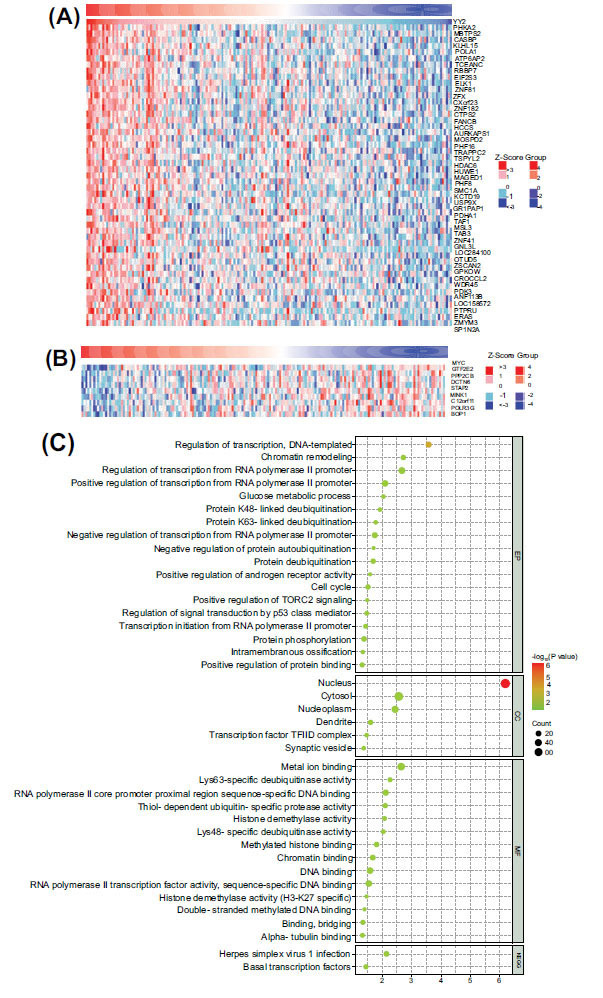
Co-expressed genes and putative functions of YY2 in ESCA. (**A**) Heatmap of the top 50 genes positively correlated with YY2 in ESCA. (**B**) Heatmap of the genes negatively correlated with YY2 in ESCA. (**C**) Functional enrichment analysis of YY2 and its co-expressed genes.

LinkedOmics analysis was employed to explore the potential functions and mechanisms of YY2 in ESCA based on TCGA-ESCA data. Pearson correlation analysis identified 166 genes positively correlated with YY2 expression (*P* < 0.01, r > 0.3) and 9 genes negatively correlated (*P* < 0.01, r < −0.3). A heatmap (Fig. **5A** and **B**) displayed the top 50 positively correlated genes and all negatively correlated genes, highlighting the extensive impact of YY2 on the transcriptome.

Gene ontology and KEGG pathway analyses of YY2 co-expressed genes revealed significant enrichment in 18 biological processes, 6 cellular components, 14 molecular functions, and 2 KEGG pathways. Notably, these genes were predominantly involved in transcriptional regulation, regulation of transcription from RNA polymerase II promoter, glucose metabolic processes, histone demethylase activity specific to H3-K27 methylation, and ubiquitin-specific protease activities (Fig. **5C**).

### 3.5 YY2 in the Glycolytic Pathway of ESCA

**Fig. (6):**
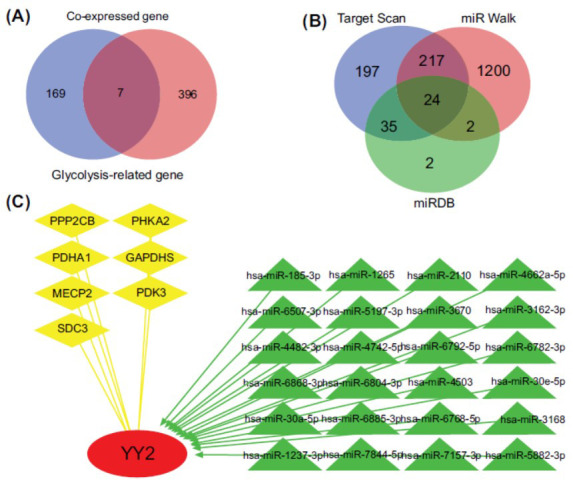
Involvement of YY2 in the glycolysis pathway in ESCA. (**A**) Venn diagram showing the overlap between genes associated with glycolysis and YY2 expression in ESCA. (**B**) Venn diagram illustrating miRNAs interacting with YY2, identified by miRwalk, miRBD, and TargetScan. (**C**) Regulatory network showing the role of YY2 in the glycolysis pathway in ESCA.

The role of YY2 in the glycolytic pathway of ESCA was investigated through a comprehensive analysis of nine glycolysis-related MSigDB gene sets, resulting in the identification of 403 genes. Among these, seven genes were found to exhibit a strong correlation with YY2 expression (Fig. **6A**). miRNA interactions with YY2 were predicted using miRwalk, miRBD, and TargetScan databases, culminating in the identification of 24 distinct miRNAs through intersection analysis (Fig. **6B**). The interactions of YY2, its co-expressed genes, and associated miRNAs in the glycolytic pathway are depicted in Fig. (**6C**).

### 3.6 Prognostic Significance of YY2 and Related Glycolytic Genes in ESCA

**Fig. (7):**
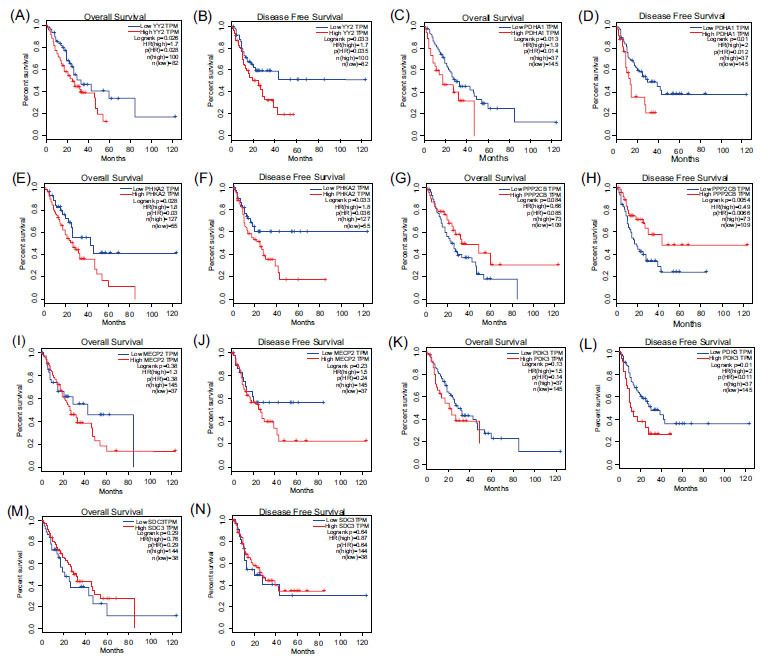
KM survival analysis of YY2 and its related glycolysis genes in ESCA. (**A-B**) Overall survival (OS) and disease-free survival (DFS) in the low and high YY2 expression groups. (**C-D**) OS and DFS in the low and high PDHA1 expression groups. (**E-F**) OS and DFS in the low and high PHKA2 expression groups. (**G-H**) OS and DFS in the low and high PPP2CB expression groups. (**I-J**) OS and DFS in the low and high MECP2 expression groups. (**K-L**) OS and DFS in the low and high PDK3 expression groups. (**M-N**) OS and DFS in the low and high SDC3 expression groups. GAPDHS was excluded due to its low expression.

GEPIA analysis revealed a significant association between high YY2 expression and decreased overall survival (OS) and recurrence-free survival (RFS) in ESCA patients (all *P* < 0.05, Fig. **7A** and **B**). Further analysis of glycolytic genes associated with YY2, including PDHA1 (Fig. **7C** and **D**), PHKA2 (Fig. **7E** and **F**), PPP2CB (Fig. **7G** and **H**), MECP2 (Fig. **7I** and **J**), PDK3 (Fig. **7K** and **L**), SDC3 (Fig. **7M** and **N**), showed that elevated expression of PDHA1 and PHKA2 was correlated with reduced OS and RFS (all *P* < 0.05). Reduced expression of PPP2CB and increased expression of PDK3 were associated with decreased RFS (all *P* < 0.05).

**Fig. (8):**
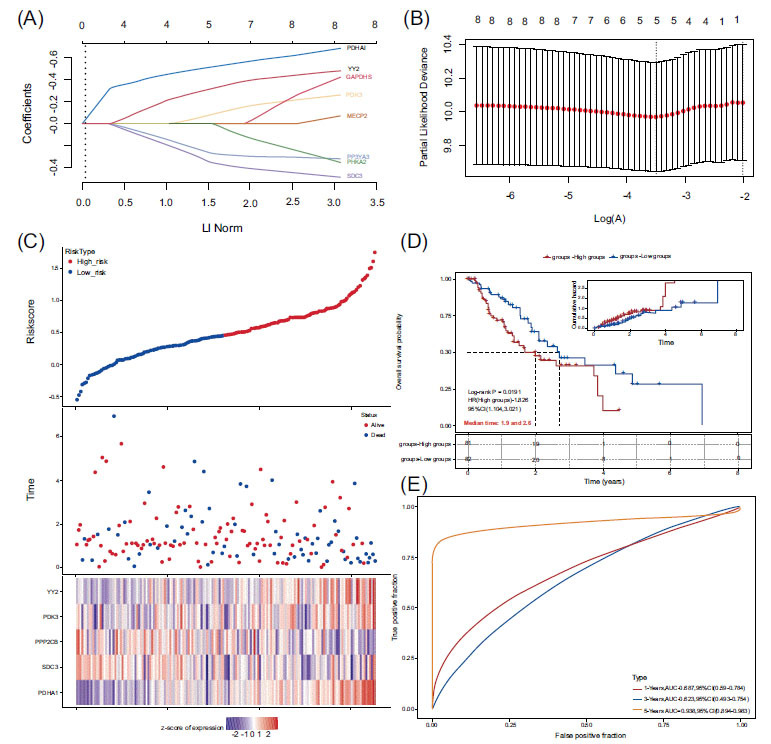
LASSO analysis of YY2 and its related glycolysis genes. (**A**) LASSO coefficient path plot. (**B**) Cross-validation curve for LASSO regression analysis. (**C**) Risk score curve, survival status, and heatmap of YY2 and its related glycolysis gene expression. (**D**) KM survival curve for the LASSO model. (**E**) ROC curves for 1-year, 3-year, and 5-year risk scores.

LASSO regression analysis of YY2 and its glycolysis-related genes produced the following gene-based prognostic model for survival risk assessment: Risk score = (0.4901) × PDHA1 + (−0.2872) × SDC3 + (−0.2209) × PPP2CB + (0.0444) × PDK3 + (0.2752) × YY2, where λ_min = 0.0302 (Fig. **8**). The Kaplan-Meier curve demonstrated significantly reduced survival times in the high-risk score group (log-rank *P* < 0.01). The ROC curve indicated that the prognostic model was highly reliable, particularly at 5 years, with an area under the curve (AUC) of 0.938 (Fig. **8**).

Analysis of the model's applicability across ESCA subtypes showed reduced survival time in EAC (log-rank *P* < 0.01), with an AUC of 0.702 at 1 year (Fig. **S2**). Although the AUC at 5 years for EAC was 0.983, overfitting was observed. The Kaplan-Meier curve showed no significant correlation between the prognostic model and survival time in ESCC (Fig. **S3**).

### 3.7 YY2 Promotes Proliferation and Migration in ECA109 and TE-1 Cells

**Fig. (9):**
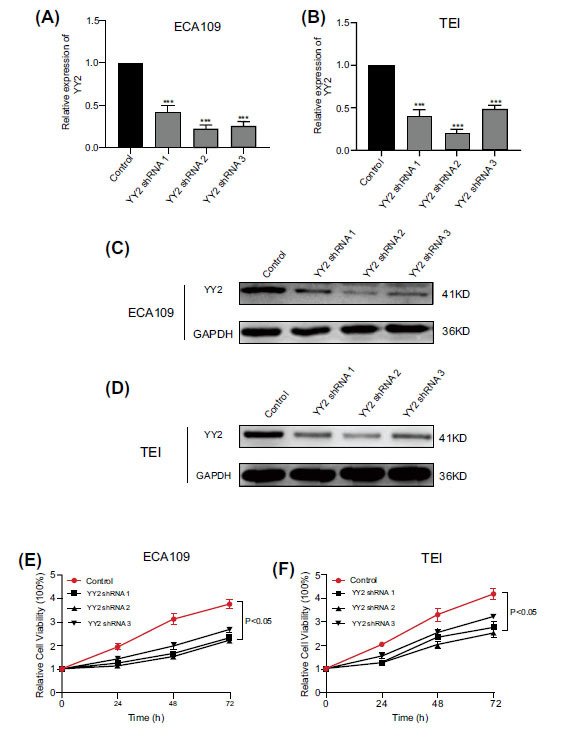
Knockdown of YY2 inhibits proliferation in ESCA. (**A-B**) Validation of shRNA-YY2 efficacy through RT-qPCR in ECA109 and TE-1 cell lines. (**C-D**) Validation of shRNA-YY2 efficacy through Western blotting in ECA109 and TE-1 cell lines. (**E-F**) Assessment of proliferation capacity in ECA109 and TE-1 cell lines using the CCK8 assay following YY2 knockdown.

**Fig. (10):**
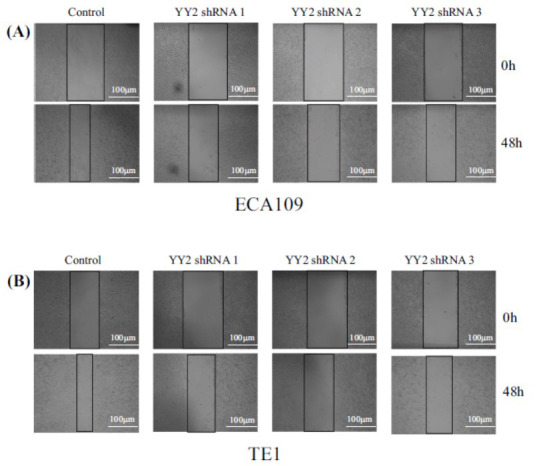
Knockdown of YY2 inhibits migration in ESCA. (**A**) Migration capacity assessed by wound healing assay in ECA109 cells. (**B**) Migration capacity assessed by wound healing assay in TE-1 cells.

To elucidate the role of YY2 in the proliferation and migration of ESCA cell lines, knockdown experiments targeting YY2 were conducted in ECA109 and TE-1 cells. The knockdown efficiency of shRNA-YY2-1, shRNA-YY2-2, and shRNA-YY2-3 was validated through RT-PCR and Western blot analyses (Fig. **9A**-**D**). The effect of YY2 knockdown on cellular proliferation was assessed using the CCK8 assay, which demonstrated a significant reduction in proliferation compared to control cells (Fig. **9E** and **F**). Additionally, a wound healing assay was performed to evaluate cell migration, revealing a marked decrease in migration following YY2 knockdown (Fig. **10**). Collectively, these findings indicate that YY2 contributes to the regulation of both proliferation and migration in ESCA cells.

## 4 DISCUSSION

The transcription factor YY2 plays a pivotal role in various tumor-associated biological processes, including cellular proliferation, metastasis, metabolic reprogramming, immune regulation, and immune surveillance [6]. This study employed bioinformatics analyses to demonstrate significant upregulation of YY2 expression in ESCA tissues relative to normal controls and revealed that elevated YY2 levels were associated with poorer OS and RFS in ESCA patients. Furthermore, the expression of YY2 was validated in our own ESCA samples, and YY2 knockdown was shown to suppress proliferation and migration in ESCA cells. These findings provide robust evidence supporting the oncogenic role of YY2 in ESCA.

Consistent with previous studies [19], YY1 has been reported to exhibit elevated expression in ESCA tissues, influencing immune cell infiltration. Moreover, siRNA-mediated knockdown of YY1 effectively suppressed the proliferation and migration of ECA109 and TE-1 cells. However, emerging evidence suggests that the tumorigenic roles of YY2 may antagonize those of YY1 [6, 7]. The zinc finger domain of YY2 shares approximately 86.4% sequence homology with YY1, indicating substantial similarity in their DNA-binding sites. The regulatory roles of YY2 and YY1 on their target genes may exhibit distinct patterns: (1) synergistic interactions between YY2 and YY1; (2) competition for occupancy at shared DNA-binding sites; or (3) regulation of unique target genes by YY2 [6, 7]. The findings of this study revealed no significant correlation between YY1 and YY2 expression in ESCA, suggesting that elevated levels of YY1 and YY2 may independently contribute to ESCA pathogenesis.

Although certain studies have highlighted its oncogenic potential [37, 38], YY2 has primarily been characterized as a tumor suppressor gene in the majority of studies [20-24]. In contrast, the oncogenic role of YY1 has been widely recognized across various cancer types [6, 16-19], though YY1 has been identified as a tumor suppressor in pancreatic cancer [39], breast cancer [40], and nasopharyngeal carcinoma [15]. Increasing evidence supports the dual functionality of specific genes, including KLF4 [41], PSME2 [42], and TGF-β [43], as both oncogenes and tumor suppressors, depending on cancer type and stage. These observations underscore the spatial and temporal dynamics of gene function, challenging simple classifications and highlighting the complexity and heterogeneity of cancer biology.

Genomic methylation abnormalities are frequently observed in tumors [44]. Hypermethylation of promoter regions often suppresses gene expression [45]. The CpG-rich YY2 promoter exhibits transcriptional suppression due to DNA methylation *in vitro*, whereas *in vivo* demethylation experiments using 5-Aza-2-deoxycytidine treatment in HEK293 cells resulted in a threefold increase in YY2 expression [46]. YY2 is methylated and demethylated at lysine 247 by SET7/9 and LSD1 *in vitro* and in cultured cells. This methylation regulates the DNA-binding capacity of YY2 and chromosomal interactions, influencing gene transcription, cellular proliferation, and tumor growth [20]. The present study identified lower methylation levels in the YY2 promoter region in ESCA tissues compared to normal tissues, although no statistically significant correlation between YY2 expression and methylation level was observed. This may be attributed to the limited sample size.

The tumor microenvironment plays a critical role in tumorigenesis. M2 macrophages and Tregs promote tumor growth by inducing immune suppression [47, 48], while B cells exhibit both anti-tumor and pro-tumor effects in solid cancers [49]. NK cells combat cancer by eliminating oncogenically transformed cells and enhancing T cell and antibody responses [50]. Immune checkpoints, as regulatory molecules of the immune system, inhibit effective anti-tumor immune responses, enabling immune evasion by tumor cells [51]. Recent studies have shown that mTORC1 upregulates B7-H3 expression through YY2, and tumors with high B7-H3 expression display an immunosuppressive phenotype characterized by reduced CD8+ T cells and activated NK cells, along with increased M2 macrophages and Tregs [37]. This study identified a positive correlation between YY2 expression and the infiltration of macrophages and B cells, as well as elevated levels of M2, NK cells, Tregs, and immune checkpoints, such as CTLA4, TIGIT, and Siglec-15 in the high YY2 expression group. These findings suggest that YY2 may promote immune suppression by increasing M2, Tregs, and immune checkpoints, potentially contributing to ESCA pathogenesis. Regarding NK cells, variations in immune algorithms and population heterogeneity may account for divergent findings. Additionally, YY2 has been implicated in modulating immunoregulatory cytokines [52, 53]. The results of this study revealed associations between YY2 expression and chemokines, immunoinhibitors, immunostimulators, and MHCs, further supporting the potential involvement of YY2 in immune responses within ESCA.

To further investigate the molecular role of YY2 in ESCA, the co-expressed genes of YY2 were analyzed, revealing their involvement in transcription regulation, methylation, glycometabolism, and ubiquitination. The current findings indicate that YY2 modulates the expression of multiple genes, and this regulation may be dosage-dependent. For instance, YY2 enhances the transcriptional activity of c-Myc at low doses but inhibits c-Myc activity at high doses [6]. In this study, a negative correlation between c-Myc and YY2 was observed, suggesting the existence of a dose-dependent relationship. Given that YY2 co-expressed genes are associated with glycometabolism and glycolysis, a critical metabolic pathway for tumor cell growth and survival, a regulatory network involving YY2 in the glycolysis pathway was constructed. This network comprises seven genes and 24 miRNAs, providing insights into the potential mechanisms by which YY2 contributes to ESCA glycolysis.

Consistent with the findings of a study by Ruan *et al.* [38], YY2 upregulation was found to be associated with unfavorable prognosis, further supporting its role as an oncogene in tumor progression. This study conducted a prognostic analysis of YY2 and its glycolysis-related genes, constructing a prognostic model that offers a novel method for predicting survival in ESCA patients. The prognostic model incorporates genes, such as YY2 [38], PDHA1 [54], SDC3 [55], PPP2CB [56, 57], and PDK3 [58, 59], all of which have been previously reported to influence cancer prognosis. The survival curves demonstrated significant associations between YY2, PDHA1, PPP2CB, and PDK3 with ESCA prognosis. Interestingly, in subgroup analyses based on pathology, the prognostic model was significantly associated with prognosis only in EAC cases. For ESCC, the reduced sample size in subgroup analyses may have limited the findings. Notably, the epidemiology [5], cellular origins [60], pathogenesis [61, 62], and molecular profiles [61, 62] of ESCC and EAC differ significantly. Although YY2 expression is elevated in both ESCC and EAC, its significance is greater in EAC. Therefore, the prognostic model may have greater clinical utility for predicting survival time and risk in EAC patients.

Considering the role of YY2 in ESCA, it emerges as a potential therapeutic target. However, no targeted drugs for the YY2 gene are currently available, highlighting an urgent need for their development. Small-molecule inhibitors remain a focal point of clinical oncology research [63, 64]. By designing small-molecule compounds that bind to YY2, its function can be inhibited, thereby interfering with ESCA progression. Drug development can be accelerated through the use of advanced technologies, including high-throughput screening [65, 66], artificial intelligence-based virtual screening [67, 68], fragment-based drug discovery [69, 70], structure-based drug design [71, 72], and DNA-encoded library screening [73].

## CONCLUSION

In conclusion, YY2 demonstrates elevated expression in ESCA, contributing to enhanced cell proliferation, migration, immune suppression, glycolysis, and unfavorable prognosis. A comprehensive regulatory network involving YY2 in glycolysis has been constructed, encompassing multiple genes and miRNAs. Additionally, a prognostic model based on YY2 and glycolysis-related genes has been developed, showing that higher risk scores are strongly associated with reduced survival rates. Collectively, these findings provide robust evidence that YY2 plays a pivotal role in ESCA pathogenesis and may serve as a valuable prognostic biomarker and innovative therapeutic target for ESCA patients.

## Data Availability

The authors confirm that the data supporting the findings of this research are available within the article.

## LIST OF ABBREVIATIONS

AUC: Area Under the Curve
BP: Biological Processes
CC: Cellular Components
DFS: Disease-Free Survival
EAC: Esophageal Adenocarcinoma
ESCA: Esophageal Carcinoma
ESCC: Esophageal Squamous Cell Carcinoma
IHC: Immunohistochemical
MF: Molecular Functions
OS: Overall Survival
PVDF: Polyvinylidene Fluoride
RFS: Recurrence-free Survival
TBST: Tris-buffered Saline with Tween-20
YY1: Yin Yang 1
YY2: Yin Yang 2
