# Supplementary material for: YY2 Serves as a Novel Prognostic Biomarker Correlated with Immune Microenvironment and Glycolysis in Esophageal Carcinoma
Source: Curr Genomics. 2025 Feb 8;26(4):312–28. doi: 10.2174/0113892029358348250124064940 (PMC12606659; doi:10.2174/0113892029358348250124064940)
Supplement: Supplementary file 1 [file CG-26-4-312_SD1.pdf]

Supplementary Material

YY2 Serves as a Novel Prognostic Biomarker Correlated with Immune Microenvironment and Glycolysis in Esophageal Carcinoma

Haimei Gou[1,2,#], Hui Yang[2,#], Jiao Cheng[2], Shuang He[2], Can Luo[2], Xin Chen[4] and Xiaowu Zhong[1,2,3,*]

[1]Department of Clinical Laboratory, Affiliated Hospital of North Sichuan Medical College, Nanchong, 637000 Sichuan, China; [2]School of Laboratory Medicine, North Sichuan Medical College, Nanchong, 637000 Sichuan, China; [3]Translational Medicine Research Center, North Sichuan Medical College, Nanchong, 637000 Sichuan, China; [4]Department of Rehabilitation Medicine, Affiliated Hospital of North Sichuan Medical College, Nanchong, 637000 Sichuan, China

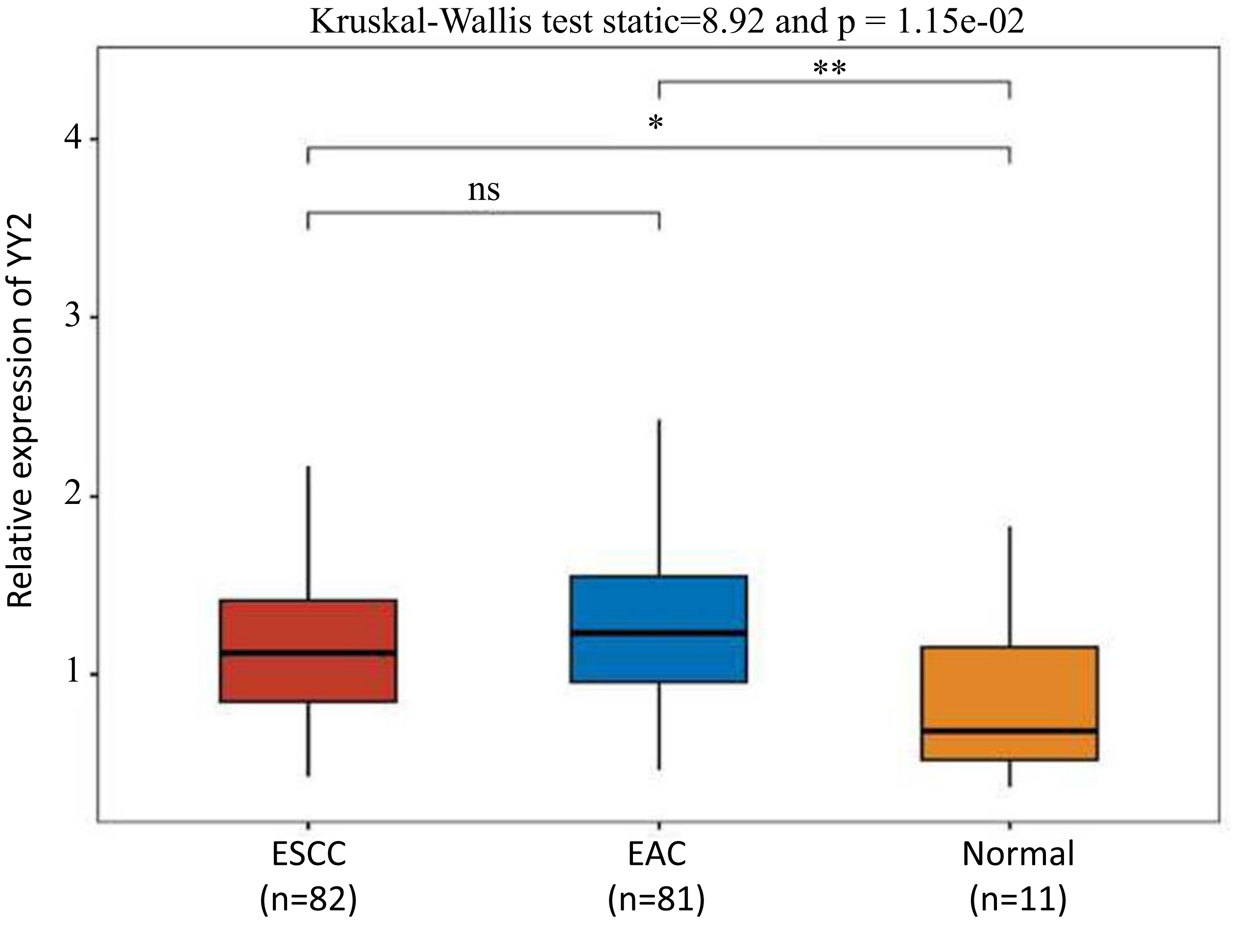

Supplementary Fig. S1. Expression levels of YY2 in ESCC tissues, EAC tissues, and normal tissues, based on TCGA data.

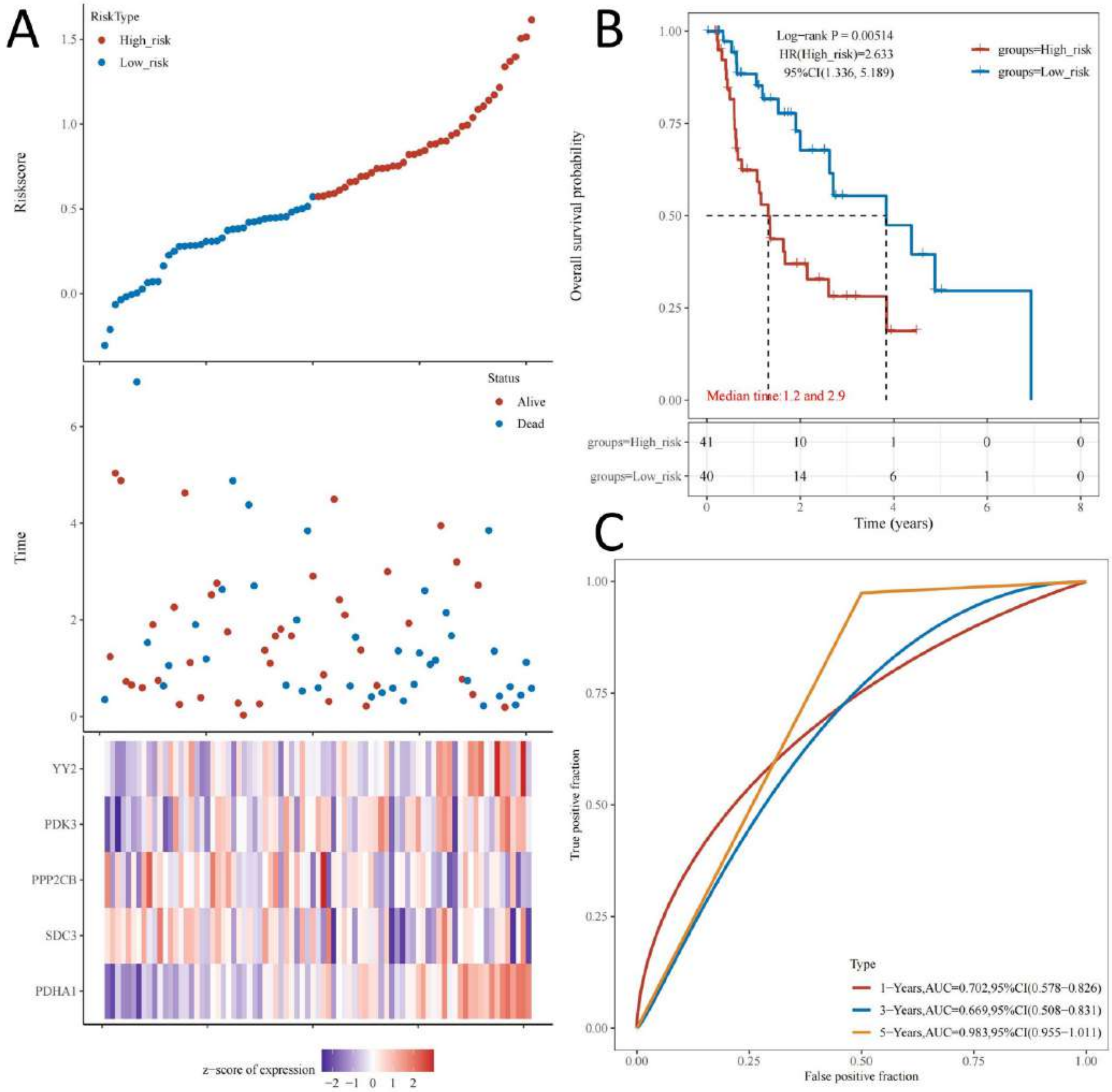

Supplementary Fig. S2. Potential clinical value of the prognostic model based on YY2 and its glycolysis-related genes in EAC. (A) Risk score curve, survival status, and heatmap of YY2 and its related glycolysis gene expression. (B) KM survival curve for the prognostic model. (C) ROC curves for risk scores.

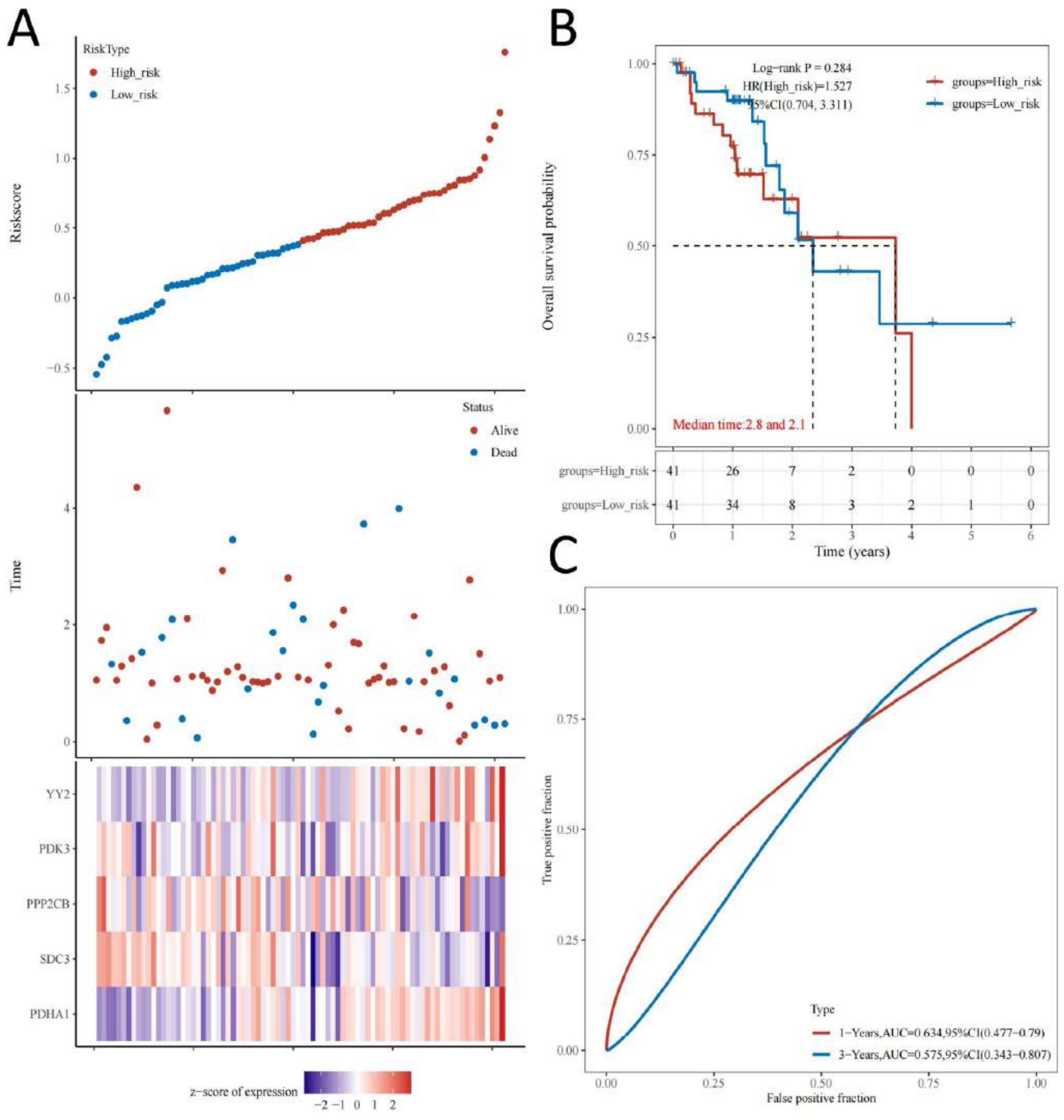

Supplementary Fig. S3. Potential clinical value of the prognostic model based on YY2 and its glycolysis-related genes in ESCC. (A) Risk score curve, survival status, and heatmap of YY2 and its related glycolysis gene expression. (B) KM survival curve for the prognostic model. (C) ROC curves for risk scores.

Supplementary Table S1. Oligonucleotide sequences and primers.

Names	Oilgonucleotides
**shRNAs**	-
YY2 shRNA1	5′-CATTGATGGTGTTGCAGCCGCTCTT-3′
YY2 shRNA2	5′-GCGACTCAGACAACCAGCTAGGCAA-3′
YY2 shRNA3	5′-CCTTGCTCTTATAGCGGCTGCGAAA-3′
**PCR primers**	-
YY2-F	5′-GCAGTGGGTGAAGGCCAGGCTG-3′
YY2-R	5′-CGGTGTGGACCAGCTGGTGTCG -3′
GAPDH-F	5′-GGAGCGAGATCCCTCCAAAAT-3′
GAPDH-R	5′-GGCTGTTGTCATACTTCTCATGG-3′

Note: F, forward primer; R, reverse primer.

Supplementary Table S2. Correlation between YY2 expression and clinical data of ESCA patients.

Clinical Features	Statistical Methods	r	*P*
years_to_birth	Spearman Correlation	-0.046	0.545
pathologic_stage	Kruskal-Wallis Test	3.784	0.286
pathology_T_stage	Kruskal-Wallis Test	5.064	0.167
pathology_N_stage	Kruskal-Wallis Test	4.307	0.230
pathology_M_stage	Wilcox Test	0.017	0.287
histological_type	Wilcox Test	-0.024	0.140
number_of_lymph_nodes	Kruskal-Wallis Test	11.250	0.590
gender	Wilcox Test	0.062	0.274
radiation_therapy	Wilcox Test	-0.012	0.838
residual_tumor	Kruskal-Wallis Test	3.440	0.179
race	Kruskal-Wallis Test	1.835	0.399
ethnicity	Wilcox Test	-0.140	0.229
